# Modelling DMC1 mediated homologous recombination repair in mouse embryonic stem cells

**DOI:** 10.3389/fcell.2026.1744837

**Published:** 2026-07-03

**Authors:** Aditya Mhaskar, Esther Sleddens-Linkels, Ivy Liang, Fiona Horne, Achmed Al-Kourdi, Johan A. Slotman, Wiggert A. Van Cappellen, Maarten W. Paul, Mehrnaz Ghazvini, Adriaan B. Houtsmuller, Alex N. Zelensky, Willy M. Baarends

**Affiliations:** 1 Department of Developmental Biology, Erasmus University Medical Center, Rotterdam, Netherlands; 2 Department of Pathology, Erasmus Optical Imaging Center, Erasmus University Medical Center, Rotterdam, Netherlands; 3 Department of Molecular Genetics, Erasmus University Medical Center, Rotterdam, Netherlands; 4 Erasmus MC iPS Facility, Erasmus University Medical Center, Rotterdam, Netherlands

**Keywords:** BRCA2, DMC1, homologous recombination, meiosis, mouse embryonic stem cells, RAD51, super-resolution microscopy

## Abstract

**Introduction:**

Programmed DNA double-strand break (DSB) formation and repair are central to meiotic homologous chromosome pairing in mammals. In meiotic cells, the DSB repair mechanism is modified compared to mitotic cells to facilitate homologous chromosome pairing. Specifically, a subset of DSBs is repaired using the homologous chromosome rather than the sister chromatid as a repair template. How the ubiquitously expressed RAD51 and meiosis-specific DMC1 recombinases function together in this process in mammals is unknown.

**Methods:**

By ectopically expressing DMC1 in somatic mouse embryonic stem (mES) cells, and analyzing its effects on RAD51-dependent homologous recombination repair, we investigated DMC1-mediated mechanistic aspects of meiotic recombination without the need for mouse models or true in vitro gametogenesis. We used ionizing irradiation to induce DSBs, performed confocal and super-resolution microscopy to study recombinase localization dynamics and regulation of its recruitment. In addition, functional assays to assess homologous recombination activity, cell cycle and cell viability were performed in the presence and absence of DMC1.

**Results:**

First, we observed that, upon ectopic expression, DMC1 was recruited to irradiation-induced DSBs in mES cells. Interestingly, in addition to BRCA2, RAD51 loading was also critical for robust DMC1 recruitment to DSBs. After initial recruitment, we observed a gradual reduction in the number of DMC1 foci over time in both live and fixed mES cells. We also found that the presence of DMC1 had no adverse effect on cell cycle progression or mES cell viability. Finally, knock-in efficiency of DMC1 expressing cells was similar to that of control cells.

**Discussion:**

We show that the presence of DMC1 in mES cells allows progression of DSB repair in a manner similar to that seen in irradiated control cells. Furthermore, the results indicate that DMC1 and RAD51 most likely form productive structures to mediate repair, while the expression of DMC1 protein neither inhibits nor stimulates homologous recombination in this mES cell model.

## Introduction

The generation of haploid gametes from diploid precursors is achieved through one round of genome duplication followed by two successive rounds of cell division in a process called meiosis. Faithful homolog segregation and genetic diversity in the resulting haploid gametes is achieved by meiotic homologous recombination and pairing between homologous chromosomes in meiotic prophase I. In addition, in many eukaryotic species, crossovers that result from homologous recombination are required to ensure faithful separation of chromosome pairs at the first meiotic metaphase-to-anaphase transition. Programmed formation of DNA double-strand breaks, and their repair by a specialized form of homologous recombination, are critical for proper meiotic prophase progression (Hunter, 2015; Zickler and Kleckner, 2015; Gray and Cohen, 2016). Unlike somatic cells, where breaks are spontaneous and random, DNA double-strand break (DSB) formation in meiosis is programmed and occurs at well-defined locations with distinct epigenetic marks (Hayashi et al., 2005; Baudat et al., 2010; Berg et al., 2010; Parvanov et al., 2010; Powers et al., 2016). Breaks are formed by the topoisomerase SPO11, in conjunction with TOPOVIBL. In this process, SPO11 remains covalently bound to the 5′ ends of DNA after break induction (Lam and Keeney, 2014). Thereafter, DSBs are resected, resulting in the removal of SPO11-bound oligos, and the thus formed ssDNA is bound by the RPA complex and meiosis-specific ssDNA binding proteins such as MEIOB and SPATA22 (Hays et al., 2017; La Salle et al., 2012; Luo et al., 2013; Souquet et al., 2013; Wold, 1997). These proteins are eventually replaced by recombinases RAD51 and meiosis-specific recombinase DMC1 (Lam and Keeney, 2014).

Similar to somatic cells, recombinase accumulation at the break sites in meiosis is thought to be BRCA2 dependent (Zelensky et al., 2014). Purified BRCA2 interacts with both RAD51 and DMC1 via 8 BRC repeat regions, with RAD51 having a higher affinity for repeats one to three, while DMC1 has a greater affinity for repeats 6–8 (Sharan et al., 1997; Jensen et al., 2010; Liu et al., 2010; Thorslund et al., 2010; Martinez et al., 2016). Further interactions between DMC1 and RAD51 and specific, slightly different FxPP motifs located within exon 14, and exon 27 of BRCA2, respectively, have been reported to have nucleofilament stabilizing effect (Thorslund et al., 2010; Kwon et al., 2023; Appleby et al., 2023; Davies and Pellegrini, 2007; Esashi et al., 2007; Dunce and Davies, 2024; Miron et al., 2024). In addition to BRCA2, its interactor PALB2 is known to be required for RAD51 recruitment in somatic cells (Xia et al., 2006), and the meiosis-specific HSF2BP-BRME1 complex has been shown to affect recombinase recruitment in mouse meiosis (Shang et al., 2020; Zhang et al., 2019; Zhang et al., 2020; Takemoto et al., 2020; Felipe-Medina et al., 2020; Brandsma et al., 2019). Finally, RAD51 interacts with DMC1 *in vitro* and is believed to facilitate foci formation *in vivo* (Lan et al., 2020; Cloud et al., 2012; Shinohara et al., 1997a; Bishop, 1994; Kurzbauer et al., 2012; Vignard et al., 2007).

In somatic HR repair, RAD51 recombinase drives the homology search and strand exchange using the sister chromatid as a repair template (Karanam et al., 2012; Hong et al., 2013; Schwacha and Kleckner, 1997). Interestingly, radiation-sensitive Rad51 mutants in budding yeast and Arabidopsis that are defective in their strand exchange activity are proficient in meiotic HR repair (Cloud et al., 2012; Hong et al., 2013; Da Ines et al., 2013). Indeed in wild-type budding yeast, RAD51 catalytic activity is downregulated in meiosis by disrupting its interaction with RAD54, an accessory factor responsible for RAD51 strand exchange activity. This is achieved via the action of Mek1 kinase which phosphorylates Hed1, which then competes with RAD54 for interaction with RAD51 (Hong et al., 2013; Callender et al., 2016; Lao et al., 2013; Busygina et al., 2008; Niu et al., 2009; Tsubouchi and Roeder, 2006; Crickard et al., 2018a; Kim et al., 2010). Furthermore, Mek1 phosphorylates RAD54 which further downregulates its interactions with RAD51 (Niu et al., 2009; Liu et al., 2014). In Arabidopsis, which lacks Hed1 and Mek1, the presence of DMC1 itself at break sites is proposed to inhibit RAD51-mediated repair (Da Ines et al., 2022). Still, complete loss of RAD51 does affect meiosis in both these species (Li et al., 2004). In mice, specific catalytic-domain recombinase mutant mouse models are not available, but depletion of either RAD51 (knockdown, or conditional knockout) or DMC1 (knockout) (Yoshida et al., 1998; Pittman et al., 1998; Dai et al., 2017; Qin et al., 2022), does interfere with the progression of meiotic prophase. Based on these observations in different species, RAD51 is considered to be an accessory factor in meiotic homologous recombination while the homology search and strand exchange function is thought to be fulfilled by DMC1. This dominant role of DMC1 is also supported by its recruitment on ssDNA proximal to the break site, where its recombinase activity is necessary for strand invasion (Slotman et al., 2020; Hinch et al., 2020). Moreover, *in vitro* experiments have shown that DMC1 recombinase has a higher tolerance towards mismatches than RAD51, which are more likely to arise when using a homologous chromosome as a repair template instead of a sister chromatid (Borgogno et al., 2016; Xu et al., 2021). This preference to use the homologous chromosome is termed the interhomolog bias, and required for recombination-mediated homologous chromosome interaction, and eventual formation of crossovers and non-crossovers. Although factors critical for ensuring interhomolog bias have been well characterized in yeast, evidence in mice is more indirect, and the exact molecular mechanisms that installs this bias remain unclear. To more easily manipulate gene and protein functions, mouse *in vitro* gametogenesis would be a very useful tool, and impressive recent advances have been made in the development of such protocols (Nosaka et al., 2025; Ishikura et al., 2021; Hikabe et al., 2016; Hayashi et al., 2017). Still, these approaches are suboptimal in terms of efficiency, costly in terms of culture requirements, and not yet fully validated in terms of normal progression of meiotic prophase (Nosaka et al., 2025; Ishikura et al., 2021; Hikabe et al., 2016; Hayashi et al., 2017). Here we take another route to allow easy functional manipulation of meiotic factors. We focus on only a single protein, DMC1, and aim to obtain insight into its meiotic functions by placing it in a somatic context. For this we chose mouse embryonic (mES) stem cells, because they are easy to maintain in culture and can readily be combined with existing microscopy techniques and be subjected to genetic manipulations. In addition, mES cells display some resemblance to primordial germ cells in terms of gene expression, with even some derepression of meiotic genes (Mise et al., 2008). For example, meiosis-specific HSF2BP, which has been shown to be critical for meiotic recombinase recruitment has been reported to be expressed in mES cells (Brandsma et al., 2019). Here, we first optimized expression of DMC1 to avoid overexpression artefacts and then proceeded to characterize DMC1 foci configurations and dynamics at repair foci in mES cells in the presence and absence of RAD51 and BRCA2. Subsequently, we assessed cellular phenotype and repair progression. The data show that in this somatic setting, the presence of DMC1 at repair sites is compatible with normal homologous recombination repair in mES cells. Our work sets the ground for further studies to investigate the nature of this repair.

## Materials and methods

### Mice

Mice were socially housed in IVC-cages with food and water *ad libitum*, in 12 hrs light and dark cycles. All mouse experiments were conducted in accordance with local regulations under the work protocols 17–867–11, 15–247–20 and 15–247–21. Adult normal male mice were euthanized to isolate testes for the generation of spermatocyte spread nuclei as described previously (Peters et al., 1997). Normal adult C57BL/6 male and female mice were used for timed matings and plugged females were killed at day 3.5 following plug detection to isolate blastocysts.

### Mouse embryonic stem (mES) cell generation

To make mES cells, 24 blastocysts (3.5 dpc) were collected and each blastocyst was placed separately on a confluent layer of irradiated mouse embryonic fibroblasts (MEFs) on 0.2% gelatin in a well of a 24 well plate with DMEM medium (Gibco, cat. no.11995065), 15% FCS (Capricorn Scientific, FBS 12-A), 100 U/mL antibiotics Penicillin-Streptomycin (Invitrogen, cat. No. 15070), 0.1 mM of Non-essential amino acids (NEAA) (Lonza, cat. No. 13–114 E), 89 µM β-mercaptoethanol (Invitrogen, 21,985–023), 1000U/mL leukaemia inhibitory factor ESGRO (Millipore, cat. No. ESG1107), 3 µM CHIR99021 (Stemgent, cat. no 04–004), one uM PD98059 (MEK inhibitor) (Cell signaling; cat. nr 9,900). The cells were incubated at 37 °C at 5% CO_2_ concentration. From passage 2, cells were cultured in medium containing 1.5 µM CHIR99021 + 0.5 µM MEK inhibitors.

### Cell culture

Male mES cell lines used in this study were generated from normal C57 B l/6 mouse blastocyst at the Erasmus MC iPS Core Facility with minor adaptations as described above (Theunissen et al., 2011). They were cultured on gelatinised (0.1% gelatin in water) cell culture dishes on top of a feeder layer of irradiated mouse embryonic fibroblast (MEFs, female). They were cultured in DMEM medium (Gibco, cat. no.11995065) supplemented with 10% FCS (Capricorn Scientific, FBS 12-A), 100 U/mL antibiotics Penicillin-Streptomycin (Invitrogen, cat. No. 15070), 0.1 mM of Non-essential amino acids (NEAA) (Lonza, cat. No. 13–114 E), 89 µM β-mercaptoethanol (Invitrogen, 21,985–023), 0.4 mM PD0325901 (Stemgent, cat. No. 04–0,006), 3 µM CHIR99021 (Stemgent, cat. No. 04–0,004), 1000 U/mL leukaemia inhibitory factor ESGRO (Millipore, cat. No. ESG1107). The cells were incubated at 37 °C at 5% CO_2_ concentration.

### Expression constructs

EGFP-T2A-DMC1 expression construct was generated by amplifying a T2A-DMC1 (mouse) fragment (gBlock, IDT) with primers WB102 and WB103 (Supplementary Table S1). The amplified T2A-DMC1 fragment and vector expressing EGFP under EF1α promoter and flanked by PiggyBac recognition sequence were excised with BglII (NEB, cat. No. R0144 S) and Acc65I (NEB, cat. No. R0599 S). Excised products were purified on an agarose gel. The purified fragments were ligated with T4 DNA ligase (NEB, cat. No. M0202 S). Cloning was verified by Sanger sequencing.

For transient DMC1 expression, EGFP sequence downstream of CAG promoter in a plasmid, was replaced with mouse DMC1 cDNA sequence. For this, mouse *Dmc1* cDNA was amplified using primers WB24 and WB25 (see below). The amplified product and plasmid expressing EGFP under CAG promoter (Addgene #11150) (Matsuda and Cepko, 2004; Miyazaki et al., 1989) were excised with EcoRI (NEB, cat. No. R3101 S) and NotI (NEB, cat. No. R3189 S). Excised products were purified on an agarose gel. The purified fragments were ligated using T4 DNA ligase (NEB, cat. No. M0202 S). Cloning was verified by Sanger sequencing.

### Cell lines

A mES line with stable mouse DMC1 expression was obtained by co-transfecting a vector containing the EGFP-T2A-DMC1 construct flanked by PiggyBac recognition sequences along with pCMV-hyPBase vector (Yusa et al., 2011). 5 days post transfection the cells were dissociated by trypsinisation and were sorted for EGFP-positive cells using FACS. The top 1%–2% of EGFP positive cells were collected and used for experiments. Transient DMC1 expression in mES cells was achieved by transfecting cells with DMC1 construct expressed under control of the CAG promoter (see above). Cells were analysed after 18 h or at said timepoints.

*Hsf2bp* ^*EGFP/EGFP*^ knock-in cells (control) and *HSF2BP* ^*EGFP/EGFP*^ *BRCA2Δ14* (BRCA2 Δ12-14) were previously published (Brandsma et al., 2019). *BRCA2* ^*Halo/Halo*^ knock-in lines were also previously generated (Paul et al., 2021).

### Quantitative real-time PCRs (qPCR) analysis

RNA from wild type and EGFP-T2A-DMC1 expressing cells and whole testis was extracted using ReliaPrep™ RNA Cell Miniprep System (Promega, cat. No. Z6010) according to the manufacturer’s instructions. Purified RNA was used to make cDNA using SuperScript™ III (Invitrogen, cat. No. 18080,093). A standard curve analysis was performed for all the primers to determine their specificity and efficiency. Primers were designed using NCBI Primer BLAST (Ye et al., 2012) (Supplementary Table S1). qPCR was performed using GoTaq® qPCR Master Mix (Promega, cat. No. A6002). qPCR data was analysed using CFX Maestro Software version 2.2 (BioRad).

### Cell cycle and apoptosis analysis

Irradiated mES cells (5 Gy) were harvested at various time points as indicated in Figure 2B. For cell cycle analysis, half of the mES cells were resuspended in 70% ice cold ethanol and were then incubated on ice for 30 mins. These cells were then centrifuged and the pellet was resuspended in PBS. The cells were again centrifuged and were permeabilized by resuspending in 400 μL PBS containing 0.1% w/v Triton X-100, 0.1 mg/mL propidium iodide (PI; Life Technologies) and 0.1 mg/mL RNAse (Roche, cat. No. 11119915,001) for 30 mins at room temperature. For apoptosis analysis, the other half of the irradiated mES cells were stained with Allophycocyanin (APC) conjugated Annexin V (Thermo Fisher, cat. No. A35110) antibody diluted in Annexin V Binding buffer (Invitrogen, cat. No. V13246) as per the manufacturers instructions. For cell cycle and apoptosis analysis, cells were analyzed and sorted using a BD FACSAria™ III cell sorter (BD Biosciences) and data were processed with FlowJo™ software v10.

### HaloPROTAC3 mediated BRCA2 degradation and siRNA mediated depletion of RAD51

mES cells knocked in with BRCA2-Halo were incubated with 300 nM HaloPROTAC3 (Promega, cat. No. GA3110) diluted in cell culture medium for 18 hrs. The cells were subsequently irradiated (5 Gy). At 60 mins post-irradiation, cells were incubated in 200 nM Halo-Ligand Janelia Fluor 646 (Promega, cat. No. HT1060) diluted in 1 mL cell culture medium for 30 mins. The cells were washed three times with PBS (37 °C) and incubated in 2 mL of fresh cell culture medium for 30 mins. Cells were further processed for immunofluorescence as described below after 2 hrs post irradiation. RAD51 depletion was performed by transfecting 300,000 mES cells with 10 nM of three dicer substrate short interfering RNA (IDT, siRNA) using Lipofectamine 2000 as described below. Cells were harvested at indicated timepoints post siRNA transfection and were processed for Western blotting. For immunofluorescence, siRNA transfected cells were seeded on laminin coated coverslips and after 24 hrs were irradiated (5 Gy). Cells were further processed for immunofluorescence as described below after 2 hrs post irradiation. Imaging was performed with a Leica SP8 confocal microscope (Leica, Mannheim) using a ×63 oil immersion objective (NA 1.40). All cells in a field of view with non-overlapping DAPI signal were analysed. BRCA2, RAD51 and DMC1 foci were counted using FIJI software (Schindelin et al., 2012) on manually thresholded images using the Analyze particle function. All statistical analyses were performed in GraphPad Prism software (version 9).

siRNA duplex sequence.

a) 5′-GCG​UCA​GCC​AUG​AUG​GUA​GAA​UCC​A-3′

3′-UUC​GCA​GUC​GGU​ACU​ACC​AUC​UUA​GGU-5′

b) 5′-GUC​AUG​GCU​AUG​CAA​AUG​CAG​CUT​G-3′

3′-GUC​AGU​ACC​GAU​ACG​UUU​ACG​UCG​AAC-5′

c) 5′-CUG​GAG​AUA​UUC​UAG​UGC​AAA​CUG​A-3′

3′-ACG​ACC​UCU​AUA​AGA​UCA​CGU​UUG​ACU-5′

### Homologous recombination mediated gene targeting at *Actb and Hsp90* locus

To test the efficiency of mCherry knock-in at the *Actb* and *Hsp90* locus, 300,000 mES cells were transfected with 5 µg of CRISPR-Cas9 guide and donor template (1:1) targeting either *Actb* and *Hsp90* locus and 0.5 µg of plasmid expressing BFP using a protocol described previously (Yao et al., 2017). mCherry positive cells were analysed 3 days after transfection using a BD FACSAria™ III cell sorter (BD Biosciences) and data were processed with FlowJo™ software v10. Knock-in efficiencies were evaluated by calculating the percentage of mCherry positive cells relative to BFP positive cells.

### Immunoblotting

Cultured mES cells were dissociated by trypsinisation and were lysed by resuspension in 100 µL of RIPA lysis buffer (Abcam, cat. No. ab156034). The cells were incubated on ice for 15 mins and the protein concentration in the cell lysate was determined on Nanodrop 2000 (Thermo Scientific) at a wavelength of 280 nm based on a standard curve generated using bovine serum albumin (Sigma-Aldrich cat. No. 126609). Protein lysate was mixed with loading buffer (4% SDS, 5% 2-mercaptoethanol, 20% glycerol, 0.004% bromophenol blue, 0.125 M Tris HCl pH 6.8) and heated at 85 °C for 10 mins. 30 μg of protein was then separated on precast 4%–15% gradient tris-acetate gels (Bio-Rad cat. No. 5671083) and transferred to nitrocellulose membrane using a semi-dry method (Trans-Blot Turbo Transfer System, Bio-Rad). Blotted nitrocellulose membranes were blocked in blocking buffer (3% milk in PBS), followed by overnight incubation in primary antibody (Supplementary Table S2) diluted in blocking buffer. Membranes were washed thrice with PBST (0.2% Tween 20) with 3% milk for 10 mins and then incubated in secondary antibody (Supplementary Table S2) diluted in blocking buffer for 1 hr in dark. Unbound secondary antibody was removed by washing the membrane thrice in PBST with 3% milk for 10 mins. The membrane was then imaged using Odyssey CLx (LI-COR Biosciences).

### Live cell imaging and FRAP

**FIGURE 1:**
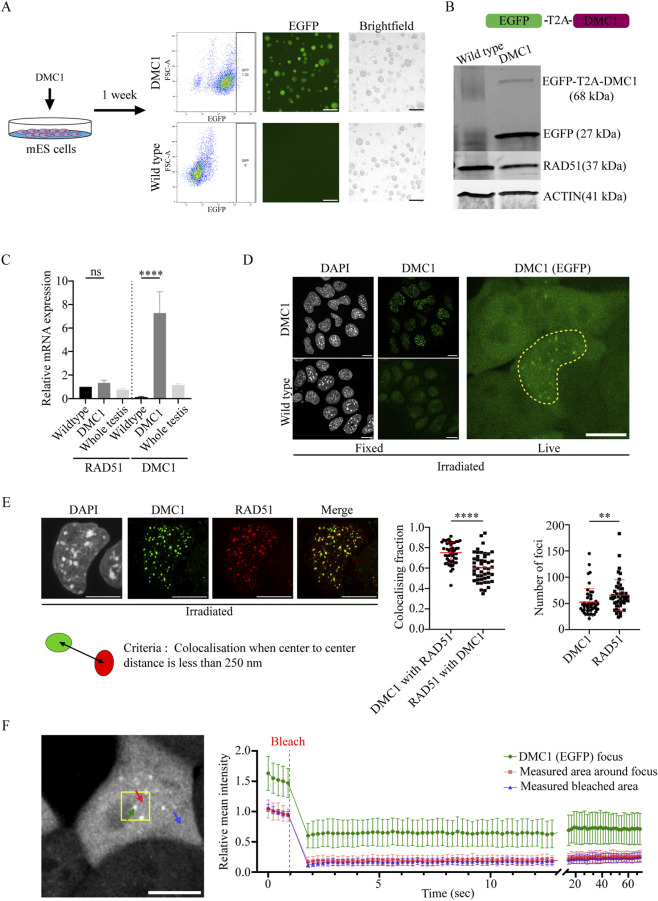
Ectopically expressed DMC1 recombinase is recruited to somatic break sites in mES cells. Stable DMC1 expression in mES cells was achieved by expressing EGFP-T2A-DMC1 construct using the PiggyBac system (see Methods). **(A)** Left panel; One week after transfection, cells with the highest EGFP signal (boxed area in the FACS plots), were FACS sorted. EGFP signal was used as a proxy to indicate DMC1 protein levels in the cells. Right panel; Images of wild type and EGFP-T2A-DMC1 expressing cells under fluorescence and bright field microscopy, scale bar represents 200 µm. **(B)** Schematic diagram of EGFP-T2A-DMC1 construct (top). Whole cell lysate was immunoblotted with antibodies against indicated proteins. 27 kDa high-intensity EGFP band indicating that the majority of EGFP-T2A-DMC1 protein is cleaved with a small fraction still being a fused protein as indicated by the weaker 68 kDa band. **(C)** Plot showing DMC1 and RAD51 mRNA expression in wildtype, EGFP-T2A-DMC1 expressing mES cells and whole testis. mRNA levels were first normalized to housekeeping gene expression and then to RAD51 levels in wild type mES cells. The data represents mean expression value ±s.d. From three independent RT-qPCR experiments. Significance is calculated using Ordinary one-way ANOVA **** = P < 0.0001 ns = non significant. **(D)** (left) Panel showing irradiated (5 Gy) nuclei of mES cells stained for DAPI (white) and anti-DMC1 (green) (right) panel showing EGFP-T2A-DMC1 foci formation post-irradiation in live cells, scale bar represents 10 µm. **(E)** Panel showing irradiated (5 Gy), EGFP-T2A-DMC1 expressing cells stained for DAPI (white), anti-DMC1 (green), anti-RAD51 (red) and merge, where yellow indicates colocalisation between RAD51 and DMC1 (middle) Quantification indicating the number of RAD51 and DMC1 foci in EGFP-T2A-DMC1 expressing mES cells 2 hrs post-irradiation (right) Quantification showing the extent of colocalisation between RAD51 and DMC1 foci. A total of n = 47 nuclei collected from two independent experiments were analyzed. Red lines indicating mean and standard deviation. Significance is calculated using a non-parametric Mann-Whitney U test assuming that the samples are unpaired. ** = P < 0.01, **** = P < 0.0001. If the detected focus in one channel displayed a center-to-center distance of <250 nm to the first nearest focus in the other channel this was defined as colocalisation (bottom left). **(F)** Panel showing screenshot of live cell imaging of a representative EGFP-T2A-DMC1 expressing cell (left) during iFRAP assay before bleaching. The area outside the yellow box was bleached. Intensity of the ROIs representing focus (green arrow in the image and line in the graph), area around the focus (black arrow in the image and line in the graph), bleached area (blue arrow in the image and line in the graph) were normalized to background intensity before bleaching and was plotted. Each data point represents normalized average intensity of ROIs of n = 22 cells from two independent experiments.

Live cell imaging (Figure 3A) and FRAP (Figure 1F) was performed on mES cells seeded on laminin (Roche, cat. No. 11243217001) coated coverslips incubated in DMEM FluoroBrite medium (Gibco, cat. No. A1896701) supplemented with serum and 2i + LIF at concentrations described previously. For live cell imaging and FRAP, cells were irradiated (5 Gy) and imaged after 30 mins or 2 hrs respectively, using Leica SP8 confocal microscope (Leica, Mannheim) with a 40 × 1.3 NA. objective in 5% CO2 atmosphere and at 37 °C. For FRAP, images were taken using 488 nm Argon laser line and emission filter set to 499–552 nm. A defined area around the EGFP-DMC1 focus in the nucleus was photobleached at 100% laser power for 0.22 sec 5 frames with 0.22 sec interval were collected before and after bleaching. An additional 50 frames with intervals of 0.22 sec were made followed by 30 more frames with a time interval of 2 sec. Images were analysed using FIJI software. ROI selections corresponding to the focus, area around the focus that falls within the unbleached area and bleached area as indicates in Figure 1F were made. Mean pixel intensities corresponding to these ROIs at all time frames pre and post bleaching were calculated using FIJI (Schindelin et al., 2012). Mean intensity relative to the average of background intensity before bleaching was computed and plotted using GraphPad Prism software (version 9). Live cell imaging was performed using 488 nm Argon laser line and emission filter set to 500–570 nm. Number of EGFP-DMC1 foci in images corresponding to said time points were counted using find maxima function in FIJI (Schindelin et al., 2012) and plotted using Graphpad Prism software (version 9).

### Immunofluorescence

Immunofluorescence staining was performed on mES cells that were seeded on laminin coated sterile 24 mm round coverslips. The coverslips were placed in six well plates and a 100 µL drop of 0.05 mg/mL solution of laminin (Roche, 11,243,217,001) was pipetted in the middle of it. The plate was left for 2 hrs in the cell culture incubator, after which the laminin solution was aspirated, and 300,000 mES cells were seeded on coverslips. The cells were irradiated (5 Gy) using the Xstrahl RS320 X-ray machine to generate DSBs. The cells were allowed to recover for 2 hrs after which they were treated for 60 sec with pre-extraction buffer (PEMT) consisting of 100 mM PIPES, 5 mM EGTA, 1 mM MgCl2, and 0.2% Triton X-100. The cells were then fixed by adding equal volume of 4% PFA in PBS (EMS, cat. no.15710) to the PEMT for 5 mins. The PEMT + PFA mix was aspirated and the cells were then incubated in 4% PFA in PBS solution for 10 mins. After fixation, the cells were washed thrice with 1 mL of PBST (0.2% Tween 20 in PBS), followed by one wash with PBS. After post fixation the cells were blocked in blocking buffer (100 mL PBS with 0.5 g Bovine serum albumin, 0.15 g Glycine) for 30 mins. In time course experiments, the blocking step was extended for each time point to enable simultaneous incubation with primary antibodies. After blocking, the coverslips were incubated overnight with 100 µL of primary antibodies (Supplementary Table S2) diluted in blocking buffer. Unbound primary antibody was removed by washing the coverslips thrice with PBST for 10 mins. They were then incubated in the dark in 100 µL of secondary antibody (Supplementary Table S2) diluted in blocking buffer for 1 hr. Unbound secondary antibody was removed by washing the coverslip with PBST for 10 mins. The coverslips were then mounted on a slide using 10 µL on Prolong Gold with DAPI (Invitrogen, cat. No. P36931) and the edges of coverslips were sealed using nail polish.

### Colocalisation analysis

For colocalisation experiments, imaging was performed with a Zeiss LSM700 Confocal Laser Scanning Microscope (Carl Zeiss, Jena), with ×63 oil immersion objective (NA 1.40). Only cells that had DMC1 foci were imaged. For experiments studying colocalisation, fluorescent Nano beads were imaged at the beginning of each session to enable correction of chromatic aberrations between images obtained at different wavelength. All images were analyzed using FIJI software. For colocalisation analysis, images in different channels were aligned using images of beads. The corrected images were then max projected and smoothened. The images were thresholded to differentiate between foci and background signal. The thresholded foci were counted in FIJI (Schindelin et al., 2012) and analysed for colocalisation using the DiAna plugin (Gilles et al., 2017). Objects in one channel with center to center distance less than 250 nm with an object in another channel were defined as colocalising. All statistical analyses were performed in GraphPad Prism software (version 9).

### STED imaging and analysis of RAD51 and DMC1 foci

STED microscopy was performed on male meiotic spreads generated as described previously (Peters et al., 1997) and on mES cells seeded on laminin coated coverslips and fixed as described above for immunofluorescence. Images were made using Leica SP8 STED (Leica, Mannheim) equipped with an 86x/1.20 water immersion objective. RAD51 (anti-RAD51, anti-rabbit Alexa Fluor 546) was imaged with a pulsed white light laser (WLL) set to 555 nm and a 660 nm CW depletion laser and emission filter set to 560–600 nm. While DMC1(anti-DMC1, anti-mouse Abberior Star 635 P) was imaged with WLL at 635 nm and a 775 nm pulsed depletion laser and emission filter set to 640–680 nm. Images were acquired with a pixel size of 20 × 20 nm. To correct for chromatic aberrations and other pixel shifts between RAD51 and DMC1 channels 100 nm TetraSpeck™ microspheres (Thermo Fisher, cat no. T7279) were included in the sample. A motorized coverslip correction collar was used for every slide to correct the lens for coverslip thickness and refractive index of the sample.

Images were processed and analyzed using FIJI software (Schindelin et al., 2012). Initially, chromatic aberration between the RAD51 and DMC1 channels along the XY axis was corrected using calibration beads. RAD51 and DMC1 foci were primarily located along the SYCP3 labelled chromosome axis in meiotic nuclei. To identify these foci, a mask was created based on the SYCP3 signal for meiotic nuclei, employing a Gaussian blur with a radius of 5. This mask was dilated by 300 nm to include foci that partially overlap with the mask. For somatic cells, a similar mask was generated using the nuclear stain DAPI. Only recombinase foci within this mask were included in the analysis. A threshold was set manually to ensure that no foci were detected outside the mask, as any signal detected in that area was likely due to the beads. A binary image was generated based on this threshold, and watershed segmentation was applied to distinguish foci that were too close together to be separated by thresholding alone. Finally, the characteristics of the foci were analyzed using the Analyse Particle function in FIJI. To reduce the background signal, only foci with an area of between 5 and 500 nm^2^ were analyzed. Various RAD51 and DMC1 configurations shown in Figure 4C were derived using analysis as described previously in (Koornneef et al., 2022).

## Results

### Meiotic recombinase DMC1 localizes to DSBs in somatic mES cells

To assess whether it is possible to observe localization of meiosis-specific recombinase DMC1 to a DSB site, we started with expressing DMC1 transiently in mES cells. For this, DMC1 encoding cDNA was cloned in a plasmid containing the CAG promoter to stimulate robust expression (see methods) and this construct was transfected in mES cells (Supplementary Figure S1A). Much like RAD51, which generates immunodetectable foci, upon recruitment to DSBs, DMC1 foci were also observed in the nuclei of mES cells following irradiation (Supplementary Figure S1B). Intriguingly, we frequently observed larger DMC1 aggregates (Supplementary Figure S1C) in particular when cells were transfected with a greater amount of plasmid DNA (Supplementary Figure S1D), indicating that this represents an overexpression artefact. Similar aggregates have been reported upon RAD51 recombinase overexpression in cells (Kachkin et al., 2022). To investigate the DMC1 foci without large cell-to-cell variation in DMC1 protein levels resulting from transient expression, we generated mES cells with stable DMC1 expression. For this, we generated a construct with the *EF-1α* promoter driving DMC1 expression that in turn, was coupled via a self-cleavage peptide to EGFP (Liu et al., 2017). The construct was integrated into the mES cell genome using the piggyBac transposon system (see Methods). Using EGFP expression as a proxy to assess DMC1 protein levels, DMC1-expressing cells were FACS sorted (Figure 1A). In yeast, both N- or C-terminal-tagged DMC1 has been reported to result in loss of function phenotype (Grishchuk et al., 2004), we verified that the majority of DMC1 expressed in mES cells was untagged, although a small fraction of EGFP-tagged DMC1 could still be detected (Figure 1B). The expressed DMC1 levels did not influence the endogenous RAD51 expression level, despite being, on average, around seven-fold higher at mRNA level (Figure 1C). Interestingly, DMC1 aggregates were no longer, or very rarely observed, and instead we observed clear nuclear DMC1 foci in almost all cells upon irradiation (compare Supplementary Figure S2A with 2B).

The majority of foci colocalised with RAD51, suggesting DMC1 recruitment to the DSB sites in mES cells (Figures 1D,E). Similar to what had been observed for yeast DMC1 (Grishchuk et al., 2004), the EGFP tagged fraction of DMC1 in mES cells was also recruited to the break sites (Figure 1D), where we speculate that it formed mixed nucleofilaments with untagged DMC1 since the latter constitutes the majority of DMC1 protein in the cells (Figure 1B). DMC1 foci numbers (visualized with anti-EGFP) exhibited a dose-dependent increase in response to irradiation, indicating robust recruitment to DNA damage sites. However, DMC1 foci numbers remained significantly lower than those of RAD51 at all tested irradiation doses (Supplementary Figure S3A,B). We also observed low numbers of DMC1 and RAD51 foci in control cells (Supplementary Figure S3A,B) confirming that DMC1 foci formation is not limited to irradiation-induced breaks. Next, we assessed whether DMC1 observed as foci is at least partially present as immobile constituent, as was previously observed for EGFP-hRAD51 in CHO40 and DT40 cells (Yu et al., 2003; Essers et al., 2002a). To this end, we performed iFRAP experiments. Our iFRAP data showed that after an initial drop in signal intensity post bleaching, corresponding to the behavior of a mobile fraction, a signal representing an immobile EGFP-DMC1 fraction is retained (Figure 1F).

### Recombinase recruitment to DSBs is dependent on both RAD51 and BRCA2

**FIGURE 2:**
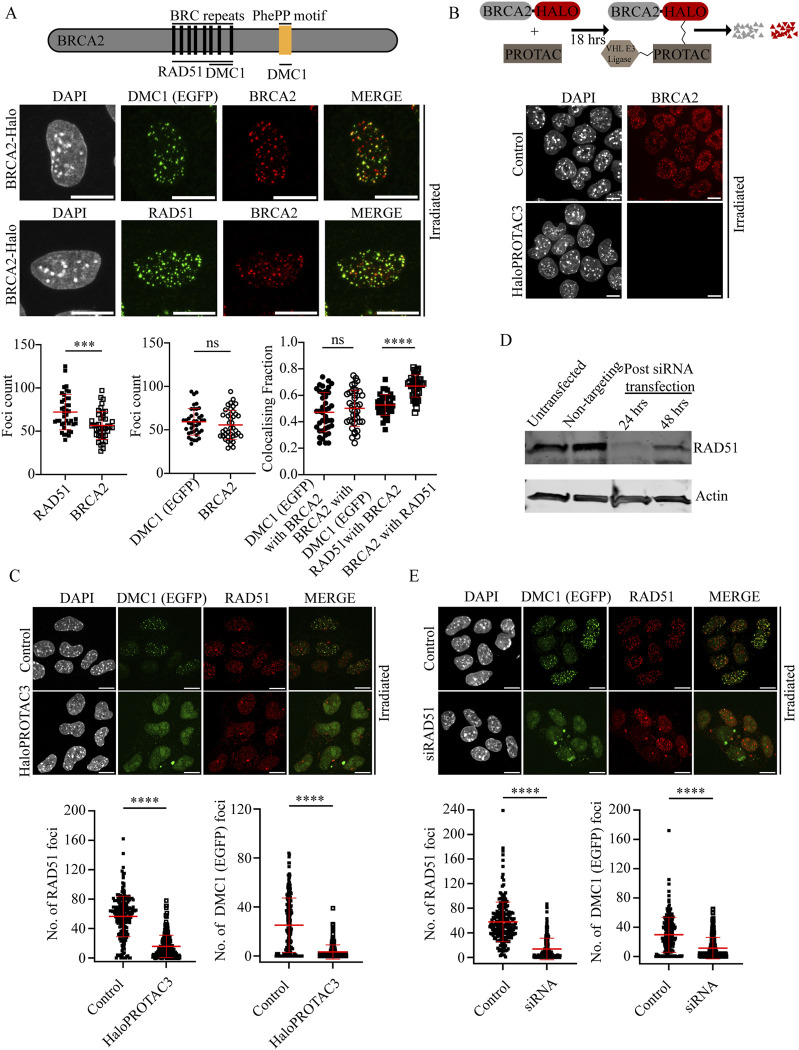
DMC1 recruitment to the break sites in mES cells depends on both BRCA2 and RAD51 recombinase **(A)** Schematic diagram of BRCA2 protein showing its RAD51 and DMC1 interaction sites (top). Panel showing representative images of irradiated (5 Gy) BRCA2-Halo expressing mES cell nuclei stained with DAPI (white), with anti-EGFP as proxy for DMC1 (green, top), with anti-RAD51 (green, bottom) and showing visualization of BRCA2-Halo with Janelia Fluor 646 (red). Quantification comparing the number of recombinase and BRCA2 foci in irradiated mES cell nuclei (bottom left). Quantification showing the extent of colocalisation between recombinase and BRCA2 as indicated in the figure (bottom right). A total of n = 40 nuclei were analyzed from two independent experiments. **(B)** Schematic representation of BRCA2-Halo degradation after 18 hrs of HaloPROTAC3 treatment (top). HaloPROTAC3 through its chloroalkane moiety forms a complex between Halo tagged BRCA2 and the VHL E3 ligase component, thus tagging Halo tag bound protein for degradation. Representative images showing loss of BRCA2-Halo (Janelia Fluor 646, red) foci in irradiated mES cell nuclei (DAPI, white) upon treatment with HaloPROTAC3 (bottom). **(C)** Panel showing the effect of BRCA2-Halo depletion on DMC1 (green, visualized with anti-EGFP) and RAD51 (red) foci formation in nuclei (DAPI, white) of mES cells (top). Quantification comparing RAD51 and DMC1 foci (visualized with anti-EGFP) in control vs. HaloPROTAC3 treated cells. > 200 nuclei from two independent experiments were analyzed (bottom). **(D)** Whole-cell lysates from conditions indicated in the figure were immunoblotted with anti-RAD51 and anti-Actin showing maximum RAD51 depletion at 24 hrs post-siRNA transfection. **(E)** Panel showing the effect of RAD51 (red) depletion on DMC1 (green, visualized with anti-EGFP) foci formation in mES cells nuclei (DAPI, white) (top). Quantification comparing RAD51 and DMC1 foci count in control vs. RAD51 depleted samples (bottom). >200 nuclei from two independent experiments were analyzed. The scale bar represents 10 µ m. Red lines indicate the mean and standard deviation. Significance is calculated using a non-parametric Mann-Whitney U test assuming that the samples are unpaired. *** = P < 0.001, **** = P < 0.0001.

DMC1 recruitment to DSBs in meiosis is believed to be BRCA2-dependent. To confirm that the DMC1 foci that we observed in somatic mES cells represent BRCA2 mediated DMC1 recruitment and/or retention, we depleted BRCA2 from EGFP-T2A-DMC1 expressing BRCA2-HaloTag knockin mES cells. First, we verified that RAD51 colocalises with BRCA2 (Figure 2A). A smaller fraction of RAD51 foci colocalised with BRCA2 than *vice versa*. This asymmetry reflects the higher RAD51 foci count compared to BRCA2, at least partly attributable to detection method differences: RAD51 signal amplification via primary and secondary antibodies versus direct Halo ligand detection of Halo-tagged BRCA2. We also observed that around 50% of BRCA2 and DMC1 foci (visualized with anti-EGFP) colocalise, suggesting their potential association at the break sites (Figure 2A). Subsequently, we depleted BRCA2 using HaloPROTAC3, which specifically degrades HaloTag fusion proteins via recruitment of the VHL E3 ligase component (Figure 2B; Supplementary Figure S4A) (Caine et al., 2020). As expected, RAD51 foci numbers are strongly decreased by BRCA2 depletion (Figure 2C). Likewise, DMC1 localization to the break sites was also impaired (Figure 2C). Interestingly, we observed normal DMC1 localization to DSBs in somatic mES cells carrying a partial *Brca2* deletion (exons 12–14) (Supplementary Figure S5A,B), that includes a region that encodes a domain that has been shown to specifically bind DMC1 (Thorslund et al., 2007), and this deletion was associated with loss of DMC1 localization in female meiosis (Koornneef et al., 2025).

Besides BRCA2, DMC1 recruitment to meiotic DSBs has been shown to be impaired in the absence of RAD51 in yeast and Arabidopsis (Lan et al., 2020; Cloud et al., 2012; Shinohara et al., 1997a; Bishop, 1994; Kurzbauer et al., 2012; Vignard et al., 2007). We knocked down RAD51 using siRNA mediated depletion. 24 hrs after transfection, the majority of RAD51 protein was depleted (Figure 2D). This was also reflected in the reduced number of RAD51 foci in the nuclei of mES cells post irradiation. Interestingly, we observed a similar reduction in DMC1 foci count (visualized with anti-EGFP) post irradiation upon RAD51 depletion in mES cells (Figure 2E).

### DMC1 accumulation at somatic break sites does not interfere with repair progression

During meiosis, several proteins including DMC1 have been shown to inhibit RAD51 strand exchange function in yeast (Hong et al., 2013; Lao et al., 2013). Ectopic expression of DMC1 has also been shown to downregulate RAD51-mediated repair in somatic cells in Arabidopsis (Da Ines et al., 2022). To test if break repair in somatic mES cells is similarly inhibited by the presence of DMC1 at the break sites, we quantified RAD51 recombinase foci over time post-irradiation. Overall, EGFP-T2A-DMC expressing cells showed a somewhat faster decline in RAD51 foci numbers as control cells, with somewhat lower numbers at 2 and 4 hrs post-irradiation, but were not different between the 2 cell lines by 6 hrs (Supplementary Figure S6A). DMC1 foci numbers (visualized by anti-EGFP) were again lower than RAD51 at each time point and also decreased over time (Supplementary Figure S6B).

**FIGURE 3:**
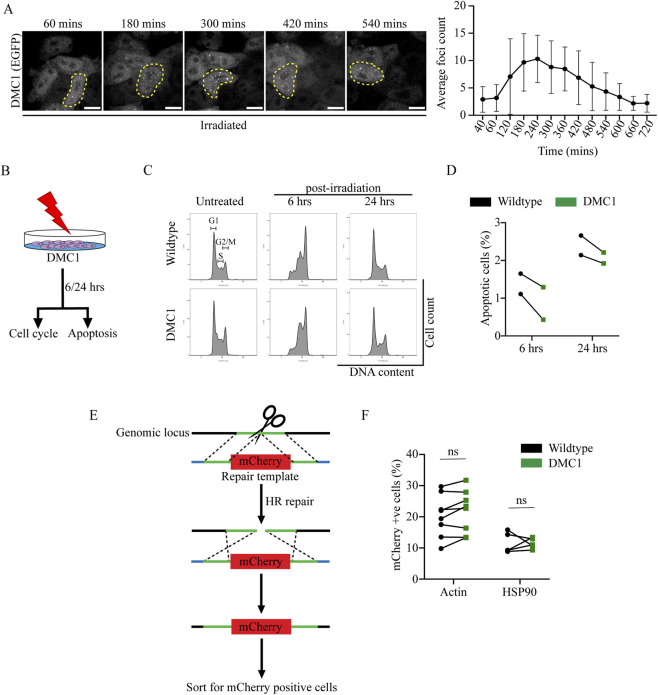
Presence of a meiotic recombinase DMC1 at break sites does not influence break repair in mES cells. **(A)** Panel showing images from indicated time points from live cell imaging of EGFP-T2A-DMC1 post-irradiation (left). Plot quantifying the number of foci from 30 nuclei from three independent experiments at different time points post-irradiation, dots representing mean foci number while lines indicate standard deviation (right). Scale bar represents 5 µm. **(B)** EGFP-T2A-DMC1 expressing mES cells were irradiated (5 Gy). Cells were then harvested at 6 hrs or 24 hrs and processed for cell cycle or apoptosis analysis as described in the Methods. **(C)** Cells stained with propidium iodide were analyzed using flow cytometry at indicated time points post-irradiation (5 Gy). **(D)** Quantification comparing the extent of cell death in EGFP-T2A-DMC1 expressing cells and WT. Data shown from two independent experiments. **(E)** Schematic depicting homologous recombination mediated mCherry integration at a genomic locus. Figure adapted from Yao et al. (2017) (see Methods). **(F)** Plots comparing mCherry knock-in efficiency at the *Actb* and *Hsp90* locus between control and EGFP-T2A-DMC1 expressing cells from n = 8 and n = 5 independent experiments respectively. Significance is calculated using Wilcoxon signed-rank test assuming that the samples are paired (pairs indicated with connecting lines). ns = non-significant.

Having a EGFP tagged DMC1 fraction allowed us to follow DMC1 foci numbers in real time. We observed a sharp increase up to 3 hrs post irradiation followed by a steady decrease until 12 hrs (Figure 3A), which is consistent with the data obtained from fixed samples. Completion of DSB repair in the presence of DMC1 at the break sites in mES cells was further supported by comparable cell cycle dynamics post irradiation between WT and EGFP-T2A-DMC1 expressing cells (Figures 3B,C). Also, we did not observe any excess cell death, which argues against these cells undergoing apoptosis following faulty repair (Figure 3D). Lastly, to directly assess the impact of DMC1 on homologous recombination repair at defined break sites, we performed a knock-in assay in which a targeted DSB was induced using a CRISPR-Cas9 approach and a homologous repair template was provided, resulting in an mCherry-T2A-tagged allele upon correct integration (Figure 3E). We applied this assay to two loci, *Actb* and *Hsp90*, in mES cells with and without stable DMC1 expression. EGFP-T2A-DMC1 expressing cells showed frequencies of mCherry-positive cells comparable to control cells lacking DMC1, indicating that homologous recombination–mediated break repair in mES cells is unaffected by DMC1 overexpression (Figure 3F).

### Super-resolution analysis of recombinase foci in somatic versus meiotic cells

**FIGURE 4:**
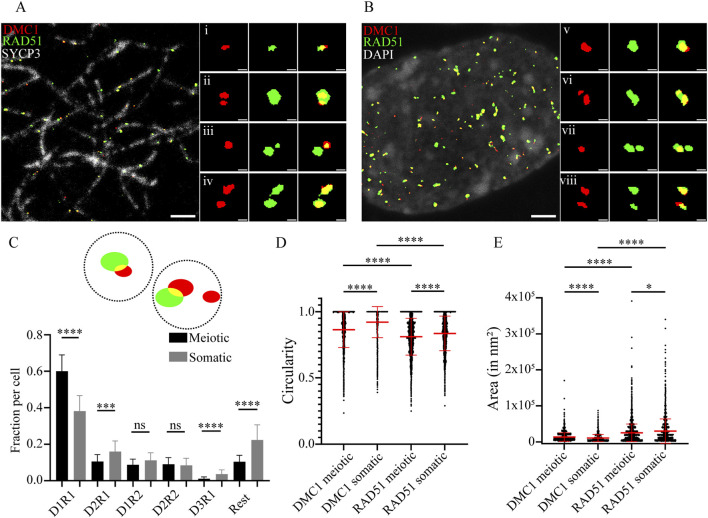
Comparison of recombinase foci between somatic and meiotic cells using STED super resolution microscopy. **(A)** Panel showing STED images of nuclear spreads of zygotene spermatocyte showing SYCP3 (white) and binary images of DMC1 (red) and RAD51 (green). Scale bar represents 2 μm. **(B)** Panel showing STED images of EGFP-T2A-DMC1 expressing somatic nuclei 2 hrs post irradiation (5 Gy) showing DMC1 (red), RAD51 (green) and nucleus (DAPI, white). i-iv show examples of various RAD51 and DMC1 configurations plotted in panel **(C)** i and v-D1R1, ii and vi-D2R1 iii and vii-D1R2, iv and viii-D2R2. Scale bar represents 0.2 μm. **(C)** Panel (top) showing schematic illustration of DMC1 (red) and RAD51 (green) foci grouped into a configuration (see Methods). Panel (bottom) distribution of RAD51 and DMC1 foci into various configurations as described in (Slotman et al., 2020). **(D)** Plot comparing circularity of RAD51 and DMC1 foci between early meiotic prophase nuclei and irradiated EGFP-T2A-DMC1 expressing mES cells. **(E)** Plot comparing the size of DMC1 and RAD51 foci between early meiotic prophase nuclei and irradiated EGFP-T2A-DMC1 expressing mES cells. A-E a total of n = 24 (meiotic) and n = 26 (somatic) cells from two independent experiments were analyzed. Significance is calculated using a non-parametric Mann-Whitney U test assuming that the samples are unpaired. ns = non-significant, **** = P < 0.0001, *** = P < 0.001, * = P < 0.1.

DMC1 recruitment to the break sites in somatic cells along with high fidelity break repair prompted us to compare RAD51 and DMC1 foci characteristics at a nanoscale resolution in somatic breaks relative to meiotic breaks. To this end, we used stimulated emission depletion (STED) microscopy to obtain super-resolution images. We generated images from meiotic spread nuclei and compared these to those obtained from somatic nuclei 2 hrs Post irradiation (Figures 4A,B). The meiotic nuclei were selected to be at the meiotic prophase stages where break induction is initiated (leptotene) and progresses (zygotene) while repair is also progressing (zygotene). In meiosis these processes are accompanied by the formation of a meiosis-specific chromosomal axis, consisting of several proteins, including SYCP3, that eventually connect in a zipper-like fashion between homologous chromosomes to form the synaptonemal complex (Zickler and Kleckner, 2015; Grey and de Massy, 2021). Axial elements start to form in leptotene and synaptonemal complex formation occurs hand-in-hand with break repair, initiating in zygotene (Zickler and Kleckner, 2015). Repair foci were selected as regions of interest (ROIs) as described in the Methods section, and binary images of nanofoci were generated in a manner similar to that described for previous analyses of RAD51 and DMC1 foci in early meiotic prophase nuclei using direct Stochastic Optical Reconstruction Microscopy (dSTORM) (Koornneef et al., 2022). Our previous data had shown that a ROI configuration with a single DMC1 and RAD51 nanofocus (termed D1R1) represents the primary form of recombinase accumulation at meiotic breaks (Slotman et al., 2020; Koornneef et al., 2022). *In vitro*, yeast RAD51 and DMC1 self-associate to form separate nucleofilaments (Lan et al., 2020; Crickard et al., 2018b), despite their ability to interact with each other. This suggests that the DMC1-RAD51 interaction is likely to be weaker compared to RAD51 or DMC1 homopolymer association with DNA, and would predict that super-resolution imaging should reveal separate RAD51 and DMC1 nanofoci.

In agreement with these meiotic and *in vitro* studies, our super-resolution analysis on early meiotic prophase spreads using STED microscopy also showed D1R1 as the most abundant recombinase configuration (Figure 4C). Although less frequent than in meiotic cells, D1R1 was also the most frequent recombinase configuration at the somatic break sites (Figure 4C). Furthermore, in both contexts, configurations with two DMC1 and a single RAD51 nanofoci (D2R1) was the second most frequently observed configuration, and in this case the frequency was higher in the somatic cells compared to meiotic cells. Additionally, more complex DxRx configurations occurred more frequently in somatic cells compared to meiotic cells (Figure 4C). Further analysis of these foci revealed that similar to meiotic foci, DMC1 foci in somatic cells were more circular than RAD51, but a bit smaller in size than their meiotic counterparts (Figures 4D,E). Taken together, super-resolution data underline robust DMC1 localization to DSB sites in somatic cells, regulated by factors also involved in DMC1 recruitment during meiosis, without compromising their capacity for high-fidelity homologous recombination repair.

## Discussion

The reported downregulation of RAD51 activity by DMC1 in meiosis along with the suggested localization of DMC1 proximal to the break sites and its tolerance towards repair templates with mismatches in vitro assays makes DMC1 a key protein in meiotic interhomologous recombination (Hinch et al., 2020; Borgogno et al., 2016; Xu et al., 2021; Lee et al., 2017; Lee et al., 2015). Here we assessed DMC1 mediated homologous recombination repair by expressing it ectopically in somatic mES cells.

### DMC1 localization to DNA damage sites in somatic cells is BRCA2 and RAD51 dependent

PiggyBac-mediated multiple genomic integrations of an EF1α-driven *Dmc1* expression cassette results in a stable overexpression system that supports robust DMC1 accumulation at DSB sites in somatic cells. The number of DMC1 foci scales with irradiation dose, indicating regulated accumulation at damage sites in response to increased break density. Moreover, DMC1 also forms foci at endogenous, most likely cell-cycle associated breaks, which are chemically distinct from irradiation-induced lesions (Cannan and Pederson, 2016). Nonetheless, DMC1 foci are consistently less numerous than RAD51 foci. This highlights the likely need for additional germ cell-specific partners to achieve DMC1 recruitment efficiencies comparable to those observed under wild-type meiotic conditions. DMC1 recruitment was further characterized by super-resolution imaging using STED microscopy. Early mouse meiotic prophase nuclei showed that recombinases exist in distinct configurations at the break sites with D1R1 (one DMC1 and one RAD51 focus) being the most abundant configuration. This agrees with previous data obtained using a dSTORM microscopy approach which also showed D1R1 to be the primary configuration *in vivo*, in particular during early meiotic prophase (Slotman et al., 2020; Koornneef et al., 2022). The theoretical resolution of dSTORM (∼20 nm) is somewhat better than that of STED (∼70 nm) (Mhaskar et al., 2021). Still, in both instances, the resolution is decreased by the primary and secondary antibody sizes used for detection by approximately 20–40 nm (Pleiner et al., 2018; Mikhaylova et al., 2015). Thus, the actual observed area sizes and shapes are most likely also influenced by the antibody complexes. Still the overall correspondence between these two datasets, and between the meiotic and somatic data sets indicates that very similar types of RAD51 and DMC1 protein accumulation patterns occur in both mES cells and spermatocytes. We observed that after 2 hrs post-irradiation the frequency of D1R1 configuration was significantly lower in somatic cells, while the frequency of D2R1 was higher as compared to meiotic cells. This might indicate that repair has progressed further in the somatic cells compared to the late leptotene-mid zygotene spermatocyte nuclei they were compared to, since for meiotic cells we previously reported a similar decrease in frequency of D1R1 with a corresponding increase in D2R1 as meiotic break repair progresses (Slotman et al., 2020). It is therefore tempting to speculate that a similar functional relationship between recombinase configurations and break repair progression also exists in somatic cells, although we note that technical differences in terms of fixation protocol, and biological differences in terms of DSBs generation and progression of repair, limit the degree to which these two results can be compared.

Loss of both RAD51 and DMC1 foci upon BRCA2 depletion, and the loss of DMC1 foci in *Rad51* knockdown mES cells provides evidence to suggests an active recruitment mechanism. BRCA2 is considered a critical mediator of DMC1 recruitment during meiosis, based both on its established role in RAD51 recruitment in somatic cells and on the absence of recombinase recruitment observed in a mouse model with gonad-specific BRCA2 deficiency (Sharan et al., 2004). BRCA2-DMC1 interaction is mediated by BRC repeats 6–8 (Martinez et al., 2016; Thorslund et al., 2007) and a so called PhePP domain encoded by exon 14 (Miron et al., 2024; Thorslund et al., 2007). The precise distribution of function between the BRC repeats and the PhePP domain, both of which interact with DMC1, remains unclear. Parallels have been drawn with the TR2 domain of BRCA2, which stabilizes RAD51 filaments, by countering the disruptive effect of BRC repeats that only interact with RAD51 monomers (Kwon et al., 2023; Davies and Pellegrini, 2007; Esashi et al., 2007; Miron et al., 2024; Esashi et al., 2005; Holthausen et al., 2011; Halder et al., 2022). Absence of the TR2 domain of BRCA2 causes increased sensitivity to DNA crosslinks, chromosomal instability, and reduced life span in mice indicating a severe defect in RAD51 function. Still the domain does not appear to be essential for meiosis, and thus may not be essential for RAD51 recruitment in that context (McAllister et al., 2002; Atanassov et al., 2005). A similar filament stabilizing effect of the PhePP domain on DMC1 has been shown *in vitro* (Dunce and Davies, 2024; Miron et al., 2024). However, mutating a critical residue in the PhePP domain (Thorslund et al., 2010), did not affect DMC1 recruitment in meiosis (Biswas et al., 2012). Based on these *in vivo* findings on TR2 and PhePP domain functions it might be suggested that their role could be redundant with other BRCA2 domains in meiosis. Recently, it has been shown by us and others that the meiosis-specific BRCA2-interactor HSF2BP is important for recombinase recruitment in spermatocytes and oocytes (Zhang et al., 2019; Brandsma et al., 2019), and by making a *Brca2* mutant where both the HSF2BP interacting domains of BRCA2 as well as the PhePP domain were deleted (Δ12-14), we observed complete loss of both RAD51 and DMC1 foci in spermatocytes, and only of DMC1 foci in oocytes isolated from this mouse mutant (Koornneef et al., 2025).

Besides BRCA2, presence of RAD51 at the break sites has been shown to be critical for DMC1 recruitment in meiosis in yeast and plants (Shinohara et al., 1997a; Bishop, 1994; Kurzbauer et al., 2012; Vignard et al., 2007; Reitz et al., 2019). The structural role of RAD51 in facilitating DMC1 recruitment is further supported by *in vitro* data (Lan et al., 2020). Evaluating DMC1 recruitment in absence of RAD51 in mice is challenging due to embryonic lethality of the complete knockout (Tsuzuki et al., 1996; Lim and Hasty, 1996). Using knockdown or conditional knockout approaches (Dai et al., 2017; Qin et al., 2022), DMC1 presence in spermatocytes was reported. However, lack of RAD51 and DMC1 co-staining data on meiotic chromosome spreads in these reports precludes assessment of RAD51 absence in cells displaying DMC1 recruitment. In our ES cell model, RAD51 was found to be crucial for DMC1 recruitment indicating possible conservation of this mechanism as observed in yeast and plants.

### Impact of DMC1 on break repair in somatic cells

Although RAD51 presence at the break site is crucial for proper DMC1 recruitment (Cloud et al., 2012; Shinohara et al., 1997a; Bishop, 1994; Kurzbauer et al., 2012; Vignard et al., 2007), its strand exchange activity is dispensable for interhomologous recombination and subsequent homolog synapsis in yeast meiosis (Cloud et al., 2012; Da Ines et al., 2013). In line with this, absence of key RAD51 cofactor RAD54 does not affect meiotic recombination in yeast, nor mice (Wesoly et al., 2006; Essers et al., 1997; Arbel et al., 1999; Bishop et al., 1999; Catlett and Forsburg, 2003; Schmuckli-Maurer and Heyer, 2000; Shinohara et al., 1997b; Hernandez Sanchez-Rebato et al., 2021; Essers et al., 2002b). In yeast, retention of RAD51 activity even tips the balance back towards somatic-like inter-sister recombination. Inhibition of RAD51 activity by Red1-Hed1-Mek1 along with chromosome axis components like Hop1 and meiosis-specific cohesin Rec8 has been extensively characterized in yeast (Hong et al., 2013; Callender et al., 2016; Busygina et al., 2008; Niu et al., 2009; Tsubouchi and Roeder, 2006; Crickard et al., 2018a; Kim et al., 2010; Liu et al., 2014; Busygina et al., 2012). Furthermore, enhanced RAD51 mediated intersister recombination in *Hed1 Dmc1* double mutants in comparison to *Hed1* alone also implicates DMC1 in suppressing RAD51 strand exchange activity (Hong et al., 2013; Lao et al., 2013). In an approach similar to ours using Arabidopsis as a model, suppression of inter-sister recombination following ectopic DMC1 expression was observed, further supporting DMC1 mediated suppression of RAD51 in meiosis (Da Ines et al., 2022). Surprisingly, ectopic expression of DMC1 in mouse embryonic stem (mES) cells did not affect homologous recombination (HR)–dependent mCherry tagging at the *Actb* and *Hsp90* loci. This observation might suggests that, in mice, the mere presence of DMC1 at DNA break sites is not sufficient to inhibit RAD51 function. Alternatively, DMC1 itself might be active and maintain HR fidelity even in the event of RAD51 inhibition. Another possibility is that both recombinases are catalytically active, or that RAD51 alone drives recombination while DMC1 remains inactive. Notably, previous studies have demonstrated that even low levels of a human RAD51 ATPase-defective mutant can interfere with endogenous RAD51 activity in mES cells (Kim et al., 2012; Forget et al., 2007; Stark et al., 2002). The somewhat faster reduction in RAD51 foci count in EGFP-T2A-DMC1 expressing cells might hint towards active contribution of DMC1 in repair, if RAD51 amount and/or activity is limiting. This interpretation, however, relies on the assumption that DMC1 forms productive nucleoprotein filaments on ssDNA analogous to those formed by RAD51. EGFP tagged recombinases have been shown to be capable of getting recruited to the break site but are defective for their strand exchange function (Grishchuk et al., 2004). Even though we observed EGFP-tagged DMC1, this represented only a small fraction of total DMC1 expressed, since the engineered self-cleavage site (T2A) mediated separation between DMC1 and EGFP for the majority of the proteins. It is likely that tagged and untagged DMC1 formed mixed nucleofilaments at the break sites. In addition, the linker which connects EGFP and DMC1 that we introduced is much longer than what has been reported for an inhibitory EGFP tag on DMC1 (Grishchuk et al., 2004). We reasoned that together, this would be sufficient to preserve DMC1 activity in mES cells. As our present data do not directly address this, the possibility that DMC1 remains catalytically inactive also cannot be ruled out.

### Concluding remarks

We have shown that DMC1 can be expressed in mES cells, without interfering with cell viability. It forms foci at irradiation induced DSBs together with RAD51, and the cells remain fully proficient in homologous recombination repair. Our readout of homologous recombination did not provide clear indications for increased recombination capacity in the presence of DMC1. We note that expression of a single meiotic protein is not sufficient, but rather provides a starting point to emulate and understand distinct meiotic homologous recombination repair. Future models might require co-expression of additional meiosis-specific factors (Zickler and Kleckner, 2015) to facilitate DMC1’s recombinase potential. Until then, this system excels for visualizing break repair dynamics in somatic cells and further dissecting regulation of DMC1 accumulation at break sites.

While expression of meiotic genes is tightly regulated, their aberrant reactivation has been reported in cancer cells (Bruggeman et al., 2018; Bruggeman et al., 2020). This includes meiotic genes such as *DMC1*, *SMC1β,* and *HORMAD1* (Bruggeman et al., 2018; Bruggeman et al., 2020; Bellio et al., 2019). In addition, cancer cell lines that express higher levels of meiotic genes demonstrated greater efficiency in break repair, suggesting a possible meiotic-like break repair mechanism in these cells (Liu et al., 2024). While the highly regulated meiotic recombination maintains genomic integrity, an unregulated meiotic-like repair in cancer cell lines would be likely to cause chromosomal translocations or deletions which could contribute to genomic instability (Feichtinger et al., 2012; Simpson et al., 2005; McFarlane and Wakeman, 2017; Gjerstorff et al., 2015). By expressing meiotic genes in our mES cell model, and by adding meiotic components sequentially, it might be possible to identify key factors that promote meiotic-like repair in somatic cells, and directly assess their effect on genomic stability. Thus, such insights may have broader implications beyond meiosis, and provide new leads for treatment of cancers for which misregulation of meiotic repair genes contributes to the phenotype.

## Data Availability

The raw data supporting the conclusions of this article will be made available by the authors, without undue reservation.
