# Supplementary material for: Modelling DMC1 mediated homologous recombination repair in mouse embryonic stem cells
Source: Front Cell Dev Biol. 2026 Jul 3;14:1744837. doi: 10.3389/fcell.2026.1744837 (PMC13376240; doi:10.3389/fcell.2026.1744837)
Supplement: Supplementary file 1 [file Image5.PDF]

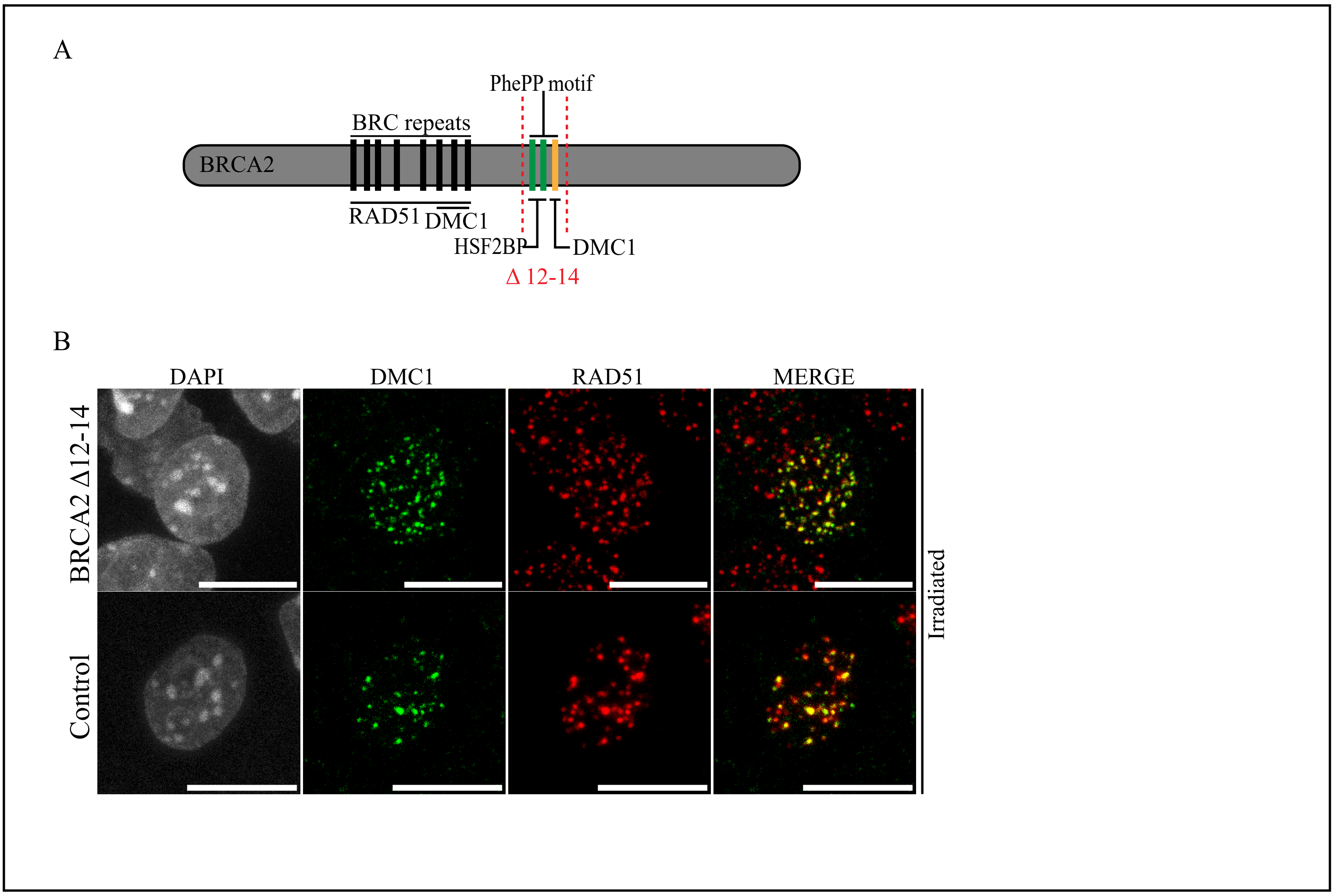

Supplemental Figure 5: BRCA2 Δ12-14 deletion does not affect DMC1 recruitment to the break sites upon transient expression.
A) Schematic showing BRCA2 Δ12-14 where HSF2BP and DMC1 binding region of BRCA2 has been deleted [69] **B)** Panel comparing recruitment of transiently expressed DMC1 (green) in control vs BRCA2 Δ12-14 mES cell nuclei in DAPI (white) along with RAD51 (red) and merge between RAD51 and DMC1 channels (yellow). Scale bar represents 10 μm.
