# Supplementary material for: Modelling DMC1 mediated homologous recombination repair in mouse embryonic stem cells
Source: Front Cell Dev Biol. 2026 Jul 3;14:1744837. doi: 10.3389/fcell.2026.1744837 (PMC13376240; doi:10.3389/fcell.2026.1744837)
Supplement: Supplementary file 2 [file Image6.PDF]

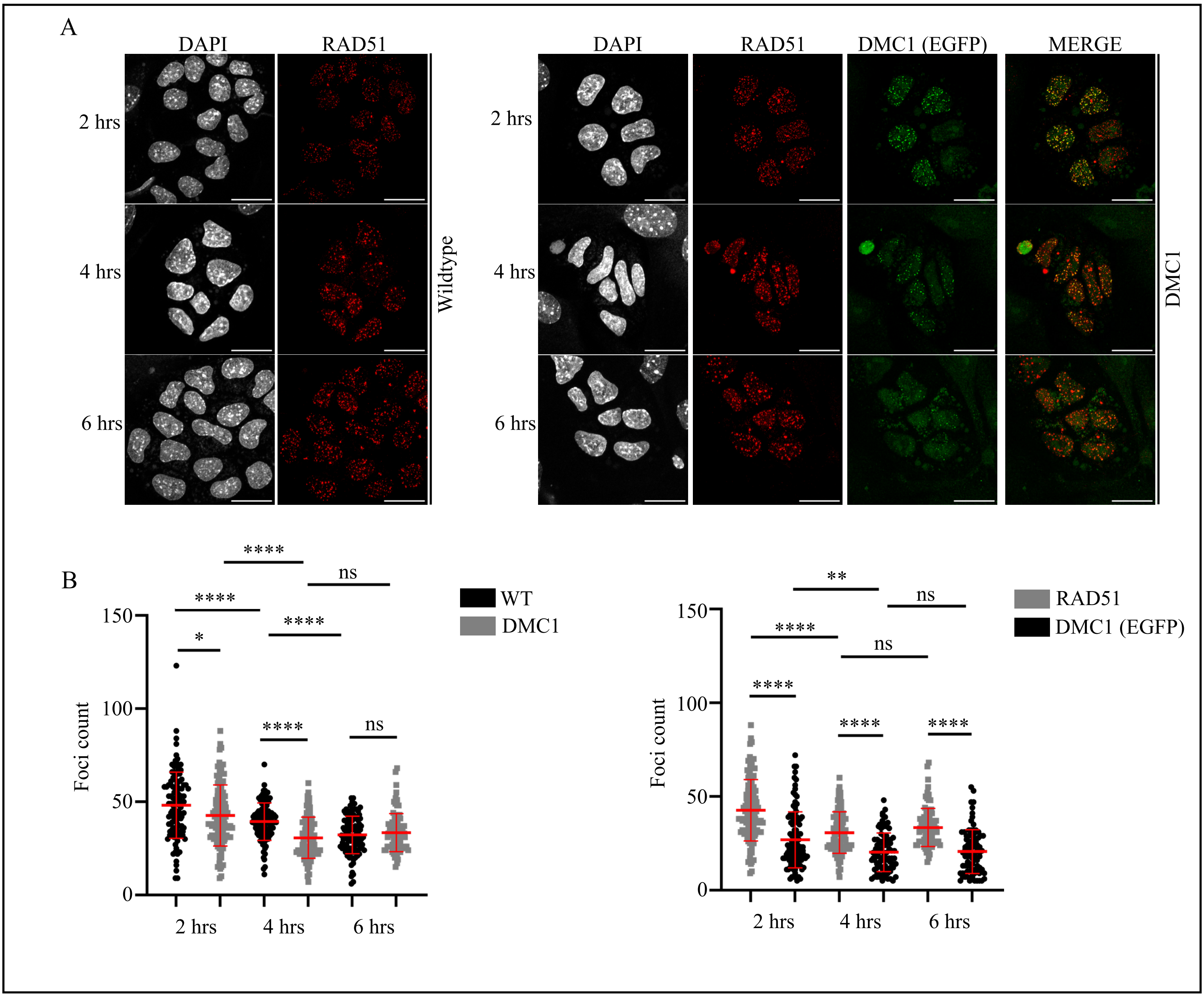

Supplemental Figure 6: EGFP-T2A-DMC1 expressing mES cells show a steady decline in recombinase foci count over time
A) Panel (left) showing RAD51 foci (red) fomation in nucleus stained with DAPI (white) in wildtype cells at various timepoints post irradiation (5 Gy). Panel (right) showing RAD51 (red) and DMC1 (green, visualised with anti-EGFP) foci formation in nucleus stained with DAPI (white) in EGFP-T2A-DMC1 expressing cells at different timepoints post irradiation (5 Gy). Scale bar represents 20 µm. **B)** Quantification (left) comparing RAD51 foci count in cells with and without EGFP-T2A-DMC1 expression at various timepoints post irradaition (5 Gy). Quantification (right) comparing RAD51 and DMC1 foci formation (visualized with anti-EGFP) in EGFP-T2A-DMC1 expressing cells at various timepoints post irradaition (5 Gy). The RAD51 foci data points (grey squares) for EGFP-T2A-DMC1-expressing cells are shared between the left and right panels. n > 100 from two independent experiments were analysed. Significance is calculated using a non-parametric Mann-Whitney U test assuming that the samples are unpaired. ns = non-significant * = P < 0.1, ** = P < 0.01, *** = P < 0.001, **** = P < 0.0001.
