# Supplementary material for: Modelling DMC1 mediated homologous recombination repair in mouse embryonic stem cells
Source: Front Cell Dev Biol. 2026 Jul 3;14:1744837. doi: 10.3389/fcell.2026.1744837 (PMC13376240; doi:10.3389/fcell.2026.1744837)
Supplement: Supplementary file 3 [file Image4.PDF]

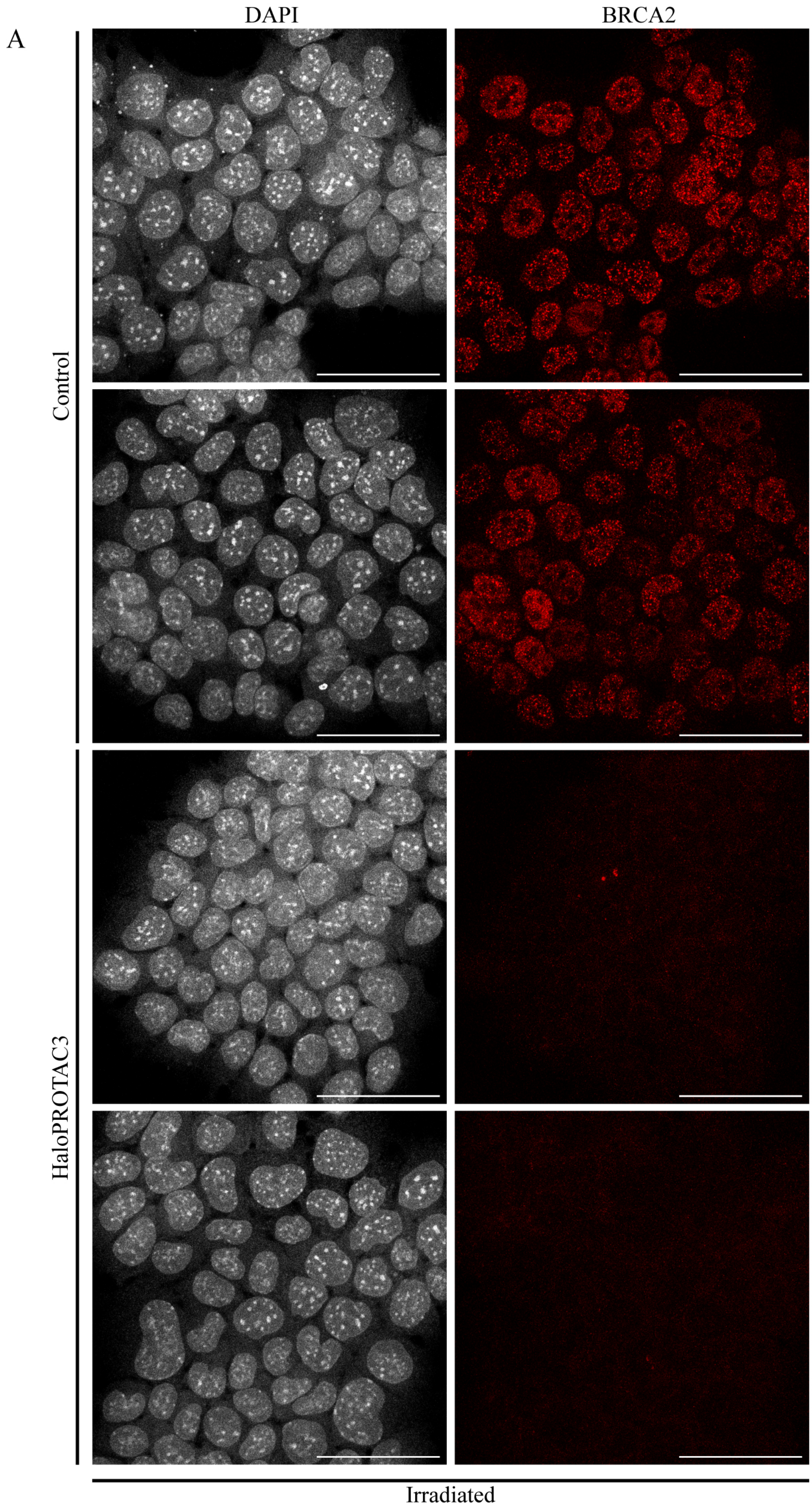

Supplemental Figure 4: Abrogation of BRCA2-Halo foci in response to HaloPROTAC3 treatment
A) Top panel showing two representative images indicating BRCA2-Halo (Janelia Fluor 646, red) foci in BRCA2$^{Halo/Halo}$ knock-in mES cell nuclei (DAPI, white) upon irradiation (5 Gy). Bottom panel showing two representative images of irradiated BRCA2$^{Halo/Halo}$ knock-in mES cell nuclei indicating loss of BRCA2-Halo foci upon HaloPROTAC3 treatment (bottom). Scale bar represents 50 µm.
