# Supplementary material for: Modelling DMC1 mediated homologous recombination repair in mouse embryonic stem cells
Source: Front Cell Dev Biol. 2026 Jul 3;14:1744837. doi: 10.3389/fcell.2026.1744837 (PMC13376240; doi:10.3389/fcell.2026.1744837)
Supplement: Supplementary file 4 [file Image2.PDF]

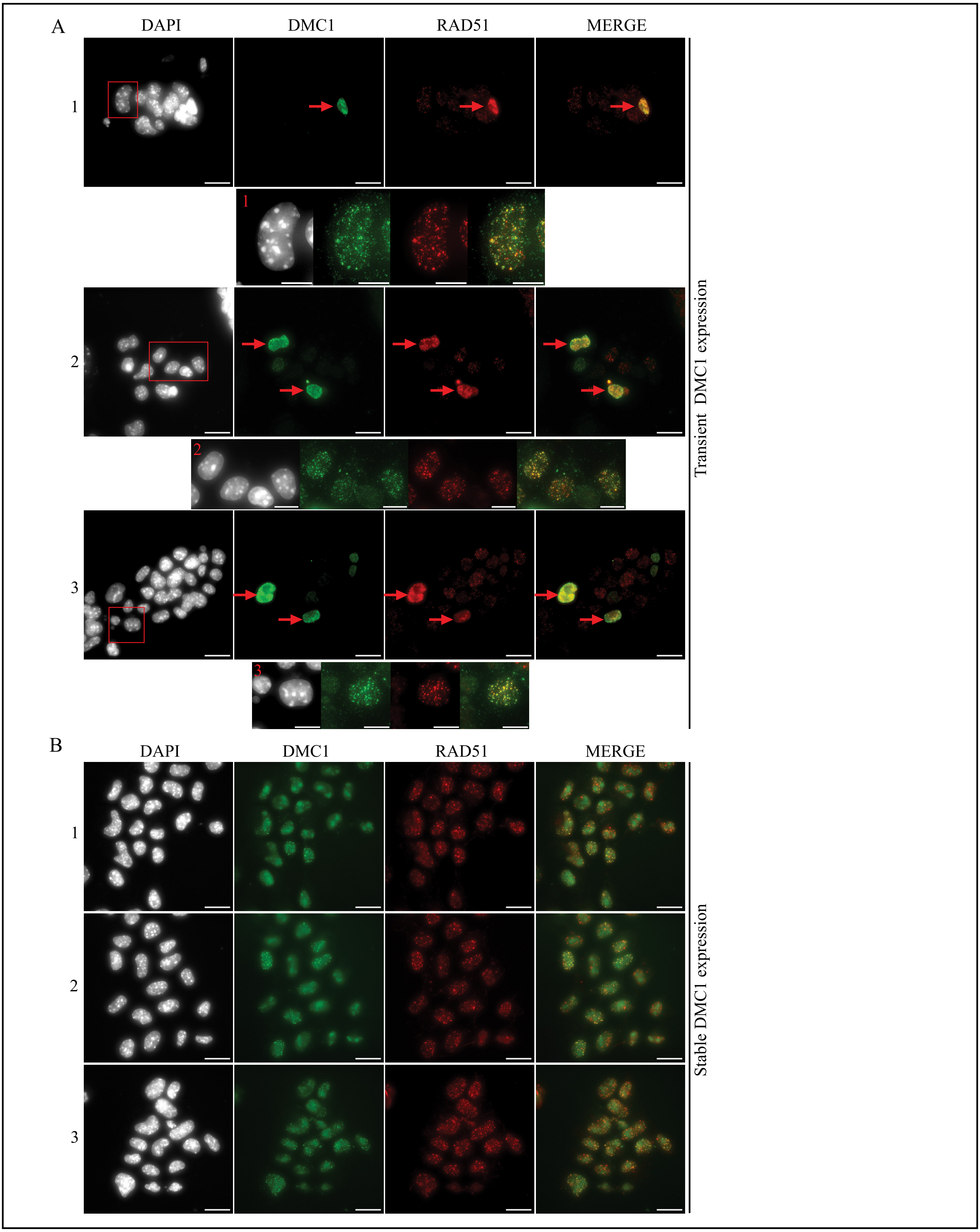

Supplemental Figure 2: No protein aggregates in mES cells with stable DMC1 expression

A) Panel showing three wide-field example image of mES cells with transient DMC1 expression showing DAPI (white), DMC1 (green) and RAD51 (red). Scale bar represents 20 µm. Cells with DMC1 aggregates are indicated by red arrows. Example cells with DMC1 foci are indicated by red squares. These cells are shown in sub-panel below have numbers (in red) and colors corresponding with the main panel but have a higher brightness and contrast settings. Scale bar represents 10 µm. **B)** Panel showing three wide-field example image of mES cells with stable DMC1 expression showing DAPI (white), DMC1 (green) and RAD51 (red). Scale bar represents 20 µm.
