# Supplementary material for: Modelling DMC1 mediated homologous recombination repair in mouse embryonic stem cells
Source: Front Cell Dev Biol. 2026 Jul 3;14:1744837. doi: 10.3389/fcell.2026.1744837 (PMC13376240; doi:10.3389/fcell.2026.1744837)
Supplement: Supplementary file 5 [file Image3.PDF]

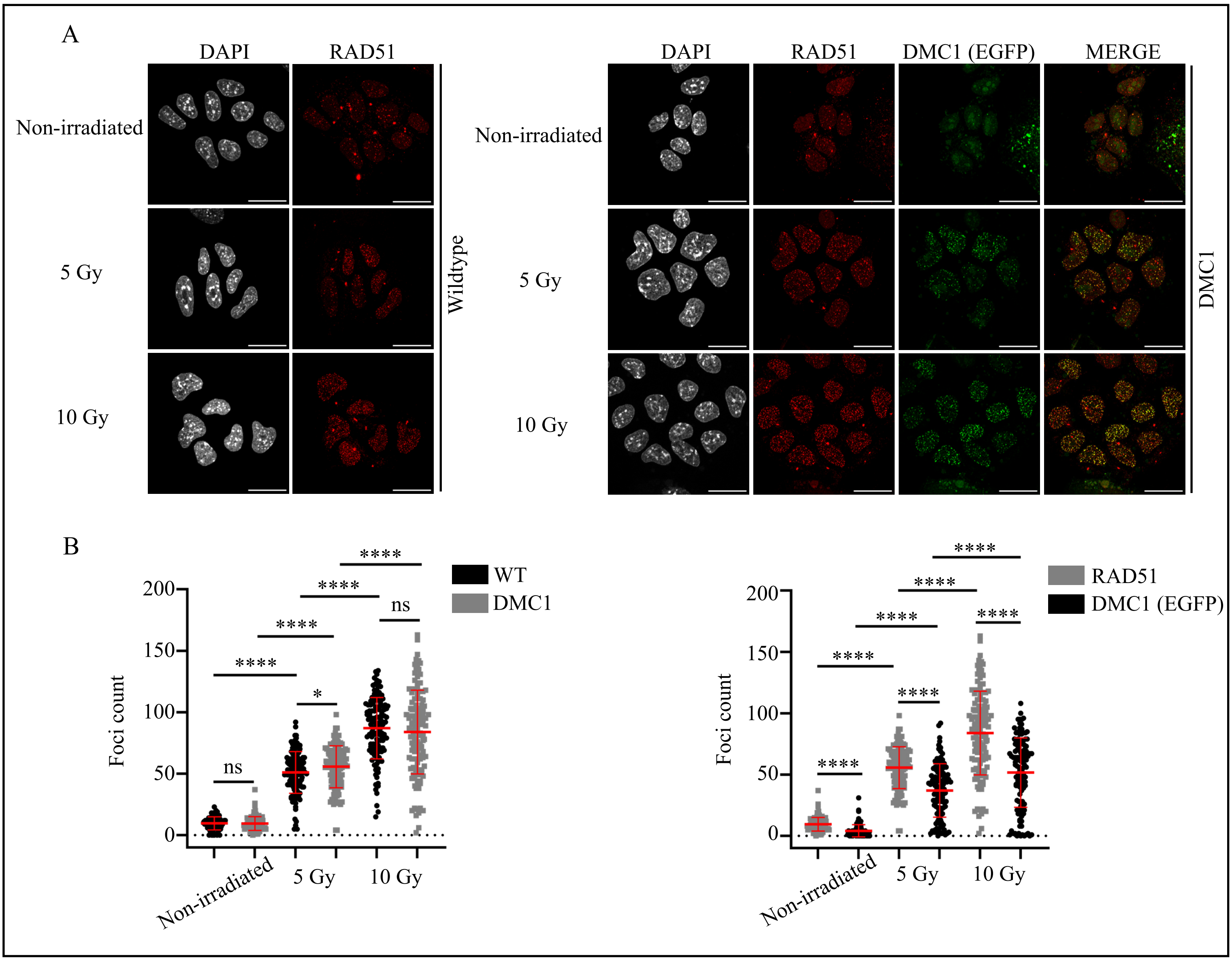

Supplemental Figure 3 : EGFP-T2A-DMC1 expressing mES cells show a dose dependent increase in recombinase foci count.
A) Panel (left) showing RAD51 foci (red) fomation in nucleus stained with DAPI (white) in wildtype cells at various irradiation dosages. Panel (right) showing RAD51 (red) and DMC1 (green, visualized with anti-EGFP) foci formation in nucleus stained with DAPI (white) in EGFP-T2A-DMC1 expressing cells in response to various irradiation dosages. Scale bar represents 20 µm. **B)** Quantification (left) comparing RAD51 foci count in cells with and without EGFP-T2A-DMC1 expression at indicated irradiation dosages. Quantification (right) comparing RAD51 and DMC1 foci formation (visualized with anti-EGFP) in DMC1 expressing cells at indicated irradiation dosages. The RAD51 foci data points (grey squares) for EGFP-T2A-DMC1-expressing cells are shared between the left and right panels. n > 100 from two independent experiments were analysed. Significance is calculated using a non-parametric Mann-Whitney U test assuming that the samples are unpaired. ns = non-significant * = P < 0.1, **** = P < 0.0001.
