# Supplementary material for: Modelling DMC1 mediated homologous recombination repair in mouse embryonic stem cells
Source: Front Cell Dev Biol. 2026 Jul 3;14:1744837. doi: 10.3389/fcell.2026.1744837 (PMC13376240; doi:10.3389/fcell.2026.1744837)
Supplement: Supplementary file 6 [file DataSheet1.PDF]

**Supplementary Table S1**

| Primer | Sequence |
|---|---|
| WB 102 | GCTAATCGAGCTATTACGGTC |
| WB 103 | TTTGTAATCCAGAGGTTGATTG |
| WB 24 | TTACATCAGAGAATTCATGAAGGAGGATCAAGTTGTGCAG |
| WB 25 | ACTTAATGATGCGGCCGCCTACTCCTTGGCATCCCCG |
| **Target** | **Sequence** |
| DMC1 | Forward- AGCTCAACTTCCAGGAACAGG<br>Reverse- TGCATAGAGCACGTTGTCCAG |
| RAD51 | Forward- GCCGGTTACCATACAGTGGA<br>Reverse- AAACTCAGTTGCCGTGGTGA |
| Actin | Forward- AACCCTAAGGCCAACCGTGAAAAG<br>Reverse- CATGGCTGGGGTGTTGAAGGTCTC |

**Supplementary Table S2**

| Primary antibodies | Host organism | Concentration |
|---|---|---|
| Anti-RAD51 (gift from R. Kanaar, Erasmus University Medical Center, Rotterdam, Netherlands) | Rabbit | 1:1000 |
| Anti-β-actin (Sigma Aldrich, cat. no. A3854) | Mouse | 1:5000 |
| Anti-GFP (Roche, cat.no. 11814460) | Mouse | 1:1000 |
| Anti-DMC1 (Abcam, cat. no. ab11054) | Mouse | 1:1000 |
| Anti-GFP 488 conjugate (Invitrogen, cat. no. A-21311) | Rabbit | 1:300 |
| Anti-GFP 488 conjugate (Chromotek, cat. no. gb2AF488) | Alpaca | 1:300 |
| **Secondary antibodies** | **Host organism** | **Concentration** |
| Anti-rabbit 680 (LICORbio, cat. no. 926-68073) | Donkey | 1:500 |
| Anti-mouse 680 (LICORbio, cat. no. 926-68072) | Donkey | 1:500 |
| Anti-mouse 800 (LICORbio, cat. no. 926-32212) | Donkey | 1:500 |
| Anti-rabbit 488 Invitrogen, cat. no. A-11008) | Goat | 1:500 |
| Anti-rabbit 546 (Invitrogen, cat. no. A-11010) | Goat | 1:500 |
| Anti-Rabbit 555 (Invitrogen, cat. no. A-21428) | Goat | 1:500 |
| Anti-rabbit 635P (Abberior, cat. no. ST635P-1002-500UG) | Goat | 1:500 |
| Anti-mouse 635P (Abberior, cat. no. ST635P-1001-500UG) | Goat | 1:500 |
