# Supplementary material for: Modelling DMC1 mediated homologous recombination repair in mouse embryonic stem cells
Source: Front Cell Dev Biol. 2026 Jul 3;14:1744837. doi: 10.3389/fcell.2026.1744837 (PMC13376240; doi:10.3389/fcell.2026.1744837)
Supplement: Supplementary file 7 [file Image1.PDF]

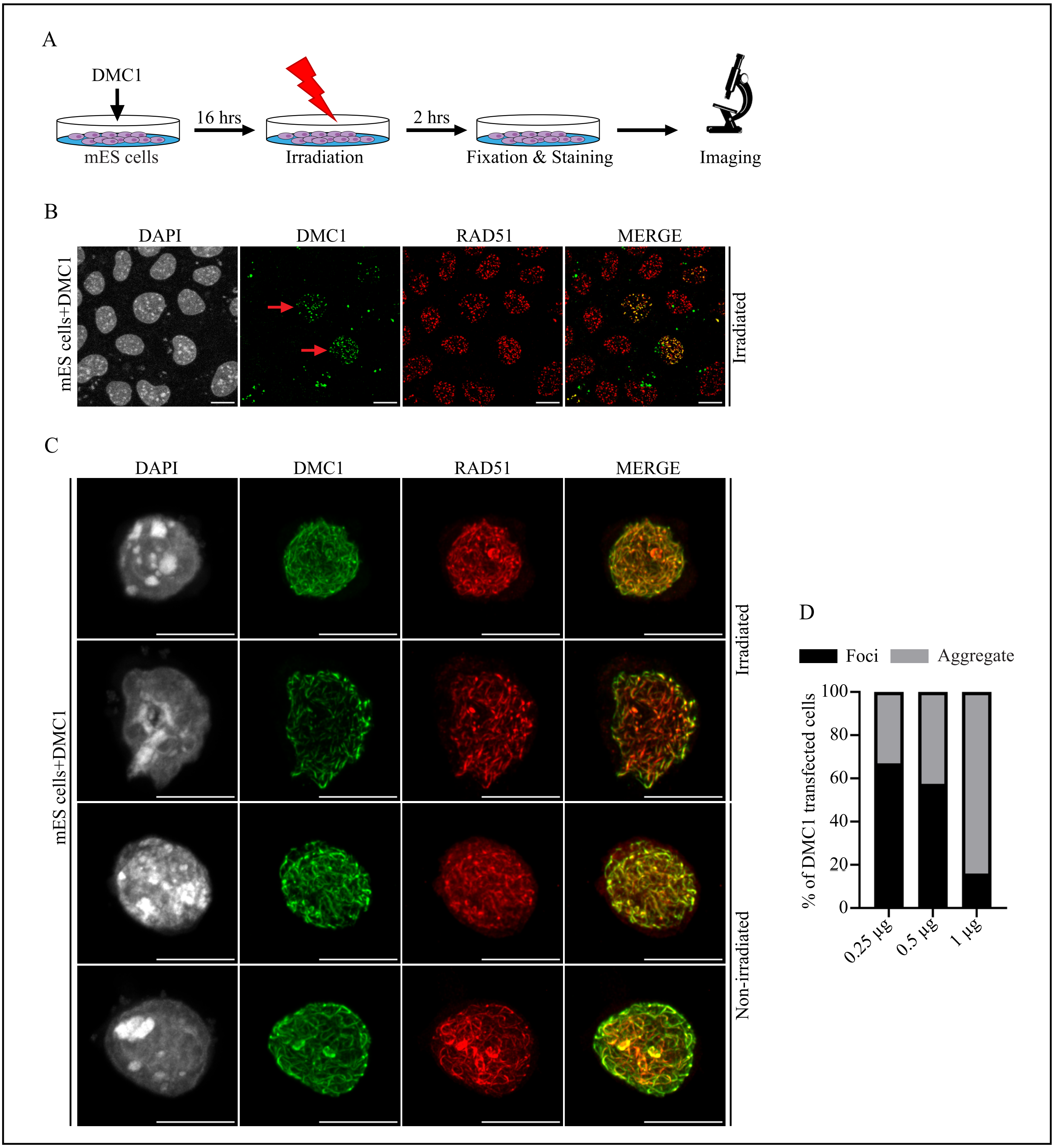

Supplemental Figure 1: DMC1 foci formation at the break sites upon transient expression is concentration dependent.
A) Schematic workflow to test DMC1 foci formation post-irradiation upon transient DMC1 expression. mES cells were transfected with DMC1 expressing plasmid. After 16 hrs, cells were irradiated (5 Gy) to induce breaks. Irradiated cells were fixed and stained after 2 hrs and were then imaged using a confocal microscope. **B)** Panel showing zoomed out image of irradiated (5 Gy) mES cells nuclei stained with DAPI (white), anti-DMC1 (green) and anti-RAD51 (red) followed by a merge between DMC1 and RAD51 channels. Arrows indicate cells expressing DMC1 (green). Scale bar represents 10 µm. **C)** Panel showing irradiated (5 Gy) and non-irradiated mES cells nuclei stained with DAPI (white) anti-DMC1 (green) and anti-RAD51 (red) followed by merge between DMC1 and RAD51 channels (yellow) showing aggregates in the nucleus. Scale bar represents 10 µm. **D)** Quantification of the percentage of mES cells having either DMC1 foci or aggregates upon transfection with 1 µg, 0.5 µg or 0.25 µg of DMC1 expressing plasmid per 500,000 cells. A total of 200 cells from two independent experiments were analysed.
